# Supplementary material for: ISG15 counteracts Listeria monocytogenes infection
Source: eLife. 2015 Aug 11;4:e06848. doi: 10.7554/eLife.06848 (PMC4530601; doi:10.7554/eLife.06848)
Supplement: Figure 4—source data 1. — Heat map of fold change of ISGylated proteins following ISG15 overexpression (in blue) and of ISGylated proteins following interferon treatment (in red). For each protein, the log2 values of the normalized M/L, normalized H/L, and normalized H/M ratios are displayed on the heat map. DOI: http://dx.doi.org/10.7554/eLife.06848.012 [file elife06848s001.pdf]

M / L	H / L	H / M	Protein ID	Protein names
11.05	11.71	1.02	P61619	Protein transport protein Sec61 subunit alpha isoform 1
7.54	7.93	0.99	P05161	Ubiquitin-like protein ISG15
5.48	4.93	0.82	Q15758	Neutral amino acid transporter B(0)
5.36	5.73	1.17	Q9NRP0	Oligosaccharyltransferase complex subunit OSTC
4.44	3.53	0.97	Q00765	Receptor expression-enhancing protein 5
4.36	5.33	1.23	P51572	B-cell receptor-associated protein 31
4.22	4.16	1.09	Q9Y6M7	Sodium bicarbonate cotransporter 3
3.91	4.65	1.09	O75915	PRA1 family protein 3
3.89	3.25	0.96	Q6DD88	Atlastin-3
3.75	4.25	1.24	Q15125	3-beta-hydroxysteroid-Delta(8),Delta(7)-isomerase
3.64	4.44	1.1	Q8TCJ2	Dolichyl-diphosphooligosaccharide--protein glycosyltransferase subunit STT3B
3.57	4.7	1.17	Q96KA5	Cleft lip and palate transmembrane protein 1-like protein
3.37	5.44	1.68	Q6MZW2	Follistatin-related protein 4
3.29	4.49	1.14	Q9UBM7	7-dehydrocholesterol reductase
3.28	3.13	0.84	Q9BTV4	Transmembrane protein 43
3.12	3.3	0.98	O15554	Intermediate conductance calcium-activated potassium channel protein 4
3.02	1.61	0.59	Q9Y282	Endoplasmic reticulum-Golgi intermediate compartment protein 3
3.02	3.87	1.18	Q9H0U3	Magnesium transporter protein 1
2.98	4.69	1.41	Q9NTJ5	Phosphatidylinositide phosphatase SAC1
2.9	4.12	1.31	Q15392	Delta(24)-sterol reductase
2.89	4.0	1.27	O15260	Surfeit locus protein 4
2.84	2.48	0.97	Q9NQC3	Reticulon-4
2.8	2.26	0.73	P61803	Dolichyl-diphosphooligosaccharide--protein glycosyltransferase subunit DAD1
2.79	3.57	1.36	Q9NZ01	Very-long-chain enoyl-CoA reductase
2.71	3.03	1.1	Q969X5	Endoplasmic reticulum-Golgi intermediate compartment protein 1
2.42	0.77	0.32	Q96I59	Probable asparagine--tRNA ligase, mitochondrial
2.37	2.67	1.0	Q9NXE4	Sphingomyelin phosphodiesterase 4
2.33	2.71	1.1	P04844	Dolichyl-diphosphooligosaccharide--protein glycosyltransferase subunit 2
2.25	1.54	0.89	Q6ZRP7	Sulfhydryl oxidase 2
2.21	1.28	0.68	P78527	DNA-dependent protein kinase catalytic subunit
2.0	2.48	1.05	O75396	Vesicle-trafficking protein SEC22b

Fold Change

0 12

M / L	H / L	H / M	Protein ID	Protein names
0.86	6.62	8.16	Q8TB05	UBA-like domain-containing protein 1
0.97	4.74	5.37	Q13325	Interferon-induced protein with tetratricopeptide repeats 5
0.71	3.95	5.11	P30508;Q29...	HLA class I histocompatibility antigen, Cw-12 alpha chain;HLA class I histocompatibility antigen, Cw-6 alpha chain;HLA class I histocompatibility antigen, Cw-14 alpha chain;HLA class I histocom...
0.43	2.39	5.04	O15533	Tapasin
0.89	3.02	3.67	O95864	Fatty acid desaturase 2
1.35	5.05	3.44	P42224	Signal transducer and activator of transcription 1-alpha/beta
0.81	3.23	3.35	Q9H0J9	Poly [ADP-ribose] polymerase 12
1.3	4.78	3.28	P41226	Ubiquitin-like modifier-activating enzyme 7
0.47	1.63	3.23	Q92626	Peroxidasin homolog
1.04	2.91	2.68	Q29940;P30...	HLA class I histocompatibility antigen, B-59 alpha chain;HLA class I histocompatibility antigen, B-55 alpha chain;HLA class I histocompatibility antigen, B-54 alpha chain;HLA class I histocompati...
1.04	2.89	2.67	P10316;P01...	HLA class I histocompatibility antigen, A-69 alpha chain;HLA class I histocompatibility antigen, A-2 alpha chain
1.1	3.04	2.56	P01891;P30...	HLA class I histocompatibility antigen, A-68 alpha chain;HLA class I histocompatibility antigen, A-74 alpha chain;HLA class I histocompatibility antigen, A-31 alpha chain;HLA class I histocompati...

Fold Change

0 12
